# Beyond maternal education: Socio-economic inequalities in children’s diet in the ABCD cohort

**DOI:** 10.1371/journal.pone.0240423

**Published:** 2020-10-13

**Authors:** Viyan Rashid, Peter J. M. Weijs, Marielle F. Engberink, Arnoud P. Verhoeff, Mary Nicolaou

**Affiliations:** 1 Department of Nutrition and Dietetics, Faculty of Sports and Nutrition, Amsterdam University of Applied Sciences, Amsterdam, The Netherlands; 2 Department Nutrition & Dietetics, Internal Medicine, Amsterdam University Medical Centers, location VUmc, Amsterdam, The Netherlands; 3 Department of Epidemiology, Health Promotion and Health Care Innovation, Public Health Service Amsterdam, The Netherlands; 4 Department of Sociology, University of Amsterdam, Amsterdam, The Netherlands; 5 Department of Public Health, Amsterdam UMC, University of Amsterdam, The Netherlands; Universidad de La Frontera, CHILE

## Abstract

**Objective:**

We examined whether the role of maternal education in children’s unhealthy snacking diet is moderated by other socio-economic indicators.

**Methods:**

Participants were selected from the Amsterdam Born Children and their Development cohort, a large ongoing community-based birth cohort. Validated Food Frequency Questionnaires (FFQ) (n = 2782) were filled in by mothers of children aged 5.7±0.5yrs. Based on these FFQs, a snacking dietary pattern was derived using Principal Component Analysis. Socio-economic indicators were: maternal and paternal education (low, middle, high; based on the highest education completed) household finance (low, high; based on ability to save money) and neighbourhood SES (composite score including educational level, household income and employment status of residents per postal code). Cross-sectional multivariable linear regression analysis was used to assess the association and possible moderation of maternal education and other socio-economic indicators on the snacking pattern score. Analyses were adjusted for children’s age, sex and ethnicity.

**Results:**

Low maternal education (B 0.95, 95% CI 0.83;1.06), low paternal education (B 0.36, 95% CI 0.20;0.52), lower household finance (B 0.18, 95% CI 0.11;0.26) and neighbourhood SES (B -0.09, 95% CI -0.11;-0.06) were independently associated with higher snacking pattern scores (p<0.001). The association between maternal education and the snacking pattern score was somewhat moderated by household finance (p = 0.089) but remained strong. Children from middle-high educated mothers (B 0.44, 95% CI 0.35;0.52) had higher snacking pattern scores when household finance was low (B 0.49, 95% CI 0.33;0.65).

**Conclusions:**

All socio-economic indicators were associated with increased risk of unhealthy dietary patterns in young children, with low maternal education conferring the highest risk. Yet, within the group of middle-high educated mothers, lower household finance was an extra risk factor for unhealthy dietary patterns. Intervention strategies should therefore focus on lower educated mothers and middle-high educated mothers with insufficient levels of household finance.

## Introduction

Maternal education is an important determinant of children’s diet, with lower education being associated with less healthy diets and dietary patterns in children [1,2]. A potential mechanism is that mother’s education reflects knowledge, attitudes and skills regarding diet and food preparation [3]. However, maternal influence on diet is not independent of other indicators of socio-economic status (SES). For example there is evidence that paternal education [4–8], household finance [5,7,9] and neighbourhood SES [8,10,11] also play a role in the dietary patterns of children.

While the role of maternal education in children’s diets has been extensively studied, there has been less consideration of how this association might be modified by other SES indicators. For instance, paternal education can also be linked to dietary patterns through knowledge and attitudes but its role in children’s diets may reflect gender roles. Household finance, on the other hand may be indicative of the potential to purchase a healthy diet [3], although it’s possible that maternal knowledge and skills can overcome financial barriers to a healthy diet. While parents are likely have a decisive role in the food consumption pattern of young children, meso-level factors such as neighbourhood SES might play an indirect role on children’s diets [8] via its influence on mothers (and fathers) via the food environment. Childhood overweight prevalence is unevenly distributed across neighbourhoods [12] and there is evidence that individuals living in low-income neighbourhoods are subject to a greater degree of food advertising [13] and have greater accessibility to unhealthy food establishments and lower accessibility to healthy food stores [14]. At the same time, there is evidence for an association between SES and food costs for diet quality [15,16].

Insight into the influence of different SES factors is relevant in helping to identify groups at higher risk of unhealthy dietary patterns. In a previous study, we observed that lower maternal education was associated with higher scores on a unhealthy snacking pattern (characterized by high intakes of sweet and savoury snacks, refined breakfast products and low intakes of whole-grain breakfast products) [17]. In this study, we wanted to investigate the additional influence of SES factors. Therefore the aim of our present study was to examine whether the role of maternal education in children’s diet is modified by other SES indicators.

## Materials and methods

### Study design and population

**Fig 1:**
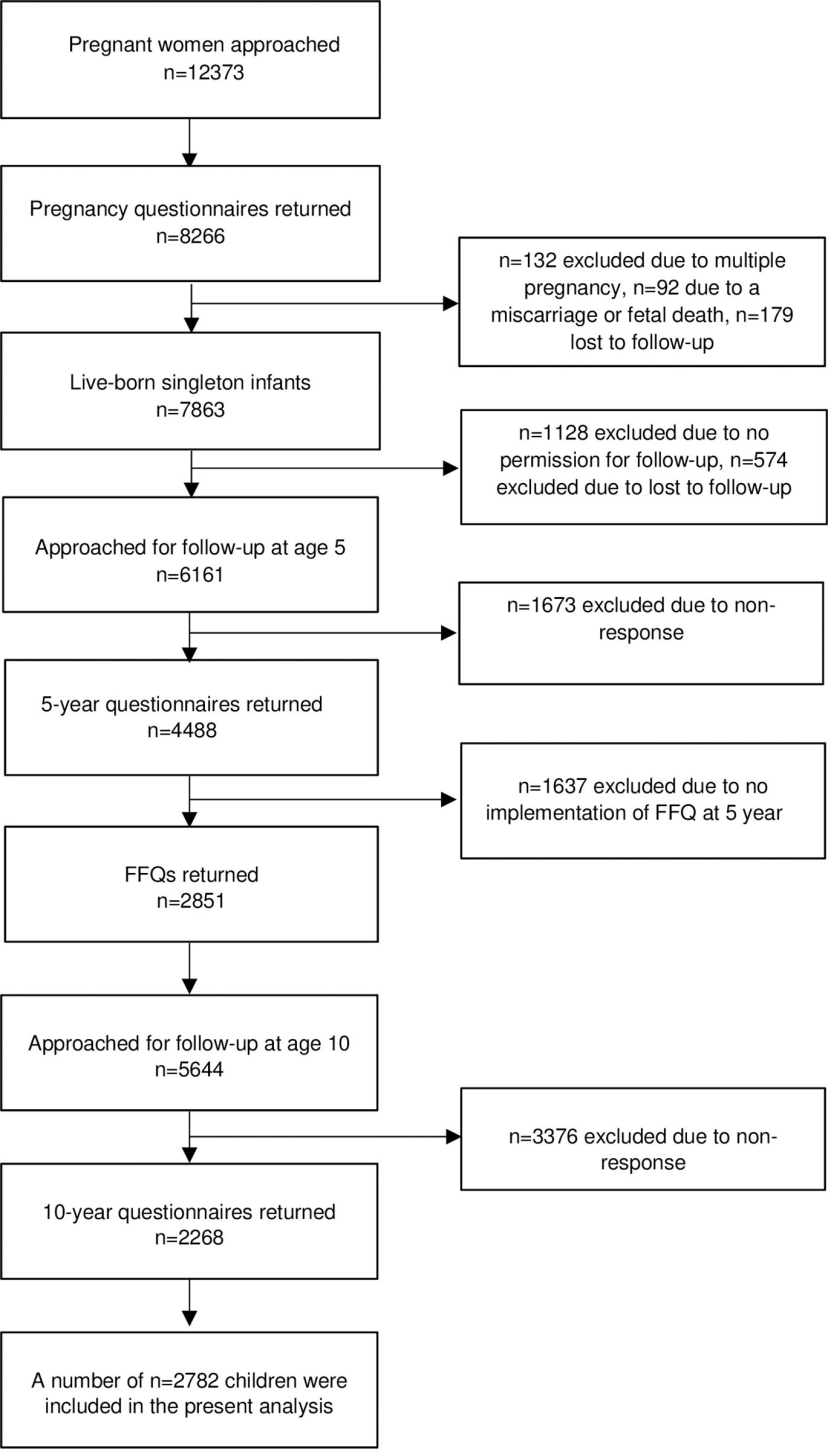
Flow-chart of the sampling procedure of the ABCD cohort (n = 2782).

Data were used from the Amsterdam Born Children and their Development (ABCD) study, a large ongoing community-based birth cohort (http://www.abcd-study.nl/). The cohort study design has been described previously [18]. Between January 2003 and March 2004, all pregnant women living in Amsterdam were invited to participate in the ABCD study. Of the 12373 women approached, 8266 women filled out a pregnancy questionnaire that was available in Dutch, English, Turkish and Arabic. A 5-year questionnaire was sent to the woman’s home address and 4488 questionnaires were filled out by the mothers. These woman’s received a self-administered Food Frequency Questionnaire (FFQ) and 2851 mothers returned the FFQ. At age 10, fathers received a 10-year questionnaire and 2268 questionnaires were returned. A number of 2782 children were included in the present analysis (Fig 1). This study was conducted according to the guidelines laid down in the Declaration of Helsinki. Approval of the study was obtained from the Central Committee on Research Involving Human Subjects in the Netherlands, the medical ethics review committees of the Academic Medical Center, Amsterdam, the VU University Medical Center Amsterdam and the Registration Committee of the Municipality of Amsterdam. All women provided written informed consent. This informed consent procedure was approved by the committees of the Academic Medical Center, Amsterdam, the VU University Medical Center Amsterdam and the Registration Committee of the Municipality of Amsterdam.

### Assessment of socio-economic factors

Data on maternal education, household finance and neighbourhood SES were collected as part of the 5-year questionnaire. Paternal education was collected as part of the 10-year questionnaire. Level of maternal and paternal education were defined as the highest education completed and were defined as: low: (a few years of) primary education/lower secondary education/lower vocational education, middle: general higher secondary education/intermediate vocational education or high: higher professional programmes or university programmes leading to a bachelor or master degree [19]. Household finance was based on the question ‘what is your current financial situation?’ and defined as low (insufficient or exactly enough) or high (ability to save money). Neighbourhood SES was determined using 2010 status scores obtained from the Social and Cultural Plan Bureau (SCP), a division of the Netherlands Institute for Social Research [20]. The children’s neighbourhoods were defined by the four digit postal code at the time of their residence at age 5. Postal code areas in Amsterdam are on average 2.5 km2 and they had a mean population of approximately 9593 residents in 2010 (interquartile range (IQR): 4530–13345) [21]. Status scores were calculated by the SCP for each postal code area using data from registries on the mean educational level, household income and employment status of area residents, analysed by Principal Component Analysis. Mean (SD) status score in the study population was 0.414 (1.18) and mean status score in Amsterdam was -0.282 (1.458) in 2010. Higher status scores represent higher SES of the neighbourhood.

### Assessment of potential covariates

Potential covariates that might influence the association between dietary patterns and SES factors were children’s age (y), sex (boys/girls) and ethnicity. Age and sex were collected via the FFQ and ethnicity via the pregnancy questionnaire. Ethnicity was based on the country of birth of the pregnant woman and her mother including both first-generation women (born outside the Netherlands) and second generation women (born in the Netherlands but whose mother was born in another country). Five ethnic categories were formed: Dutch, African Surinamese, Turkish, Moroccan and other ethnicities (mainly non-western origin).

### Assessment of diet

At age 5, mothers filled in a validated 71-item FFQ developed by TNO Food (Zeist, The Netherlands) [22]. In brief, per food item, consumption frequency, portion size and the type of product consumed over the last 4 weeks was reported by the mother of the child. Frequency options were “never”, “less than once a week”, “once a week”, “2–3 times a week”, “4–5 times a week”, and “6–7 times a week”. Food items were assessed in units (e.g. a piece of fruit and a slice of bread) and in household units (e.g. a glass and a tablespoon). Intake of items such as breakfast cereals, vegetables or pasta were asked in standard tablespoons which could give a reliable idea of the actual eaten portion size for children. Dietary patterns were derived in a previous study using Principal Component Analyses (PCA) [17]. In that study four dietary patterns were identified: snacking; full-fat; meat; and healthy, explaining 21% of the variation in dietary intake. In the current study we focused on children’s intake of the snacking pattern.

## Statistical analysis

Population characteristics were described in number, percentages or means with standard deviations (SD). ANOVA and Post-hoc Bonferroni were used to show differences by maternal education group. Non-parametric correlation by Spearman was used to check for multicollinearity between maternal education and other variables (paternal education 0.436, household finance 0.292, neighbourhood SES 0.295, snacking pattern score -0.381, age -0.109, sex 0.011 and ethnicity -0.341) and for correlations between paternal education and other variables (household finance 0.219, neighbourhood SES 0.215, snacking pattern score -0.249, age -0.021, sex 0.035 and ethnicity -0.186). Multiple linear regression analysis with listwise deletion was used to assess the association between maternal education (n = 2766) and paternal education (n = 1803), household finance (n = 2738), and neighbourhood SES (n = 2752) on the snacking pattern score (n = 2782). The individual snacking pattern score was used as continuous dependent variable and SES factors (maternal and paternal education level, household finance and neighbourhood SES) used as independent variables. Model 1 describes the association between maternal education and the snacking pattern score. Subsequently, in model 2, 3 and 4, one individual SES factor (resp. paternal education, household finance or neighbourhood SES) was added. In model 5, all SES factors were added simultaneously, to examine the association of all SES factors combined on the snacking pattern score. All models were adjusted for child’s age, sex and ethnicity. Effect modification between maternal education and paternal education, household finance and neighbourhood SES was tested by multiple linear regression analysis and considered potentially significant at a p-value<0.10. Additionally they were tested for heterogeneity by sex by including an interaction term of sex with each of the SES factors. No effect modification was found indicating that the association between maternal education and SES factors on the snacking pattern score, were similar in boys and girls. Statistical analyses were performed in SPSS version 22 for windows and the level of statistical significance was set at 0.01.

## Results

### Population characteristics

Population characteristics based on maternal education level are presented in Table 1. Children with lower educated mothers had significantly more often lower educated fathers, lower household finance, lower neighbourhood SES and higher snacking pattern scores than children from middle-high and higher educated mothers, reflecting higher intakes of snack items.

**Table 1:** Socio-economic characteristics of the study population by maternal education (n = 2766).

Population	Population by maternal education n = 2766					
Characteristics	low n = 287 (10.4%)		middle-high n = 552 (20.0%)		high n = 1927 (69.6%)	
**Age, in years**						
Mean, SD	5.83	(0.52)	5.75	(0.50)	5.67	(0.47)*
**Sex**						
Boys, n (%)	154	(50.7)	280	(50.7)	980	(50.9)
**Ethnicity**						
Dutch, n (%)	140	(48.8)*	390	(70.7)*	1750	(90.8)*
Surinamese, n (%)	23	(8.0)*	58	(10.5)*	35	(1.8)*
Turkish, n (%)	31	(10.8)*	20	(3.6)*	9	(0.5)*
Moroccan, n (%)	48	(16.7)*	42	(7.6)*	20	(1.0)*
Other ethnicities, n (%)	45	(15.7)*	42	(7.6)*	113	(5.9)*
**Paternal education**						
Low, n (%)	51	(44.0)*	42	(13.8)*	48	(3.5)*
Middle, n (%)	39	(33.6)*	118	(38.7)*	181	(13.1)*
High, n (%)	26	(22.4)*	145	(47.5)*	1149	(83.4)*
**Household finance**						
Low, n (%)	191	(68.0)*	243	(44.7)*	468	(24.5)*
High, n (%)	90	(32.0)*	301	(55.3)*	1443	(75.5)*
**Neighbourhood SES**						
Mean, SD	-0.727	1.24*	-0.069	1.28*	0.500	1.15*
**Snacking pattern score**						
Mean, SD	0.992	1.11*	0.329	1.01*	-0.246	0.84*

Maternal education was based on the highest education completed. Increasing neighbourhood SES scores represent higher neighbourhood SES and positive snacking pattern scores indicate higher consumption of food groups in that pattern.

* Sign (P<0.01) is significant with all groups and based on ANOVA and Post-hoc Bonferroni.

### SES indicators, including maternal education, in relation to the snacking pattern

The associations between maternal education, paternal education, household finance, neighbourhood SES and the snacking pattern score are presented in Table 2. Maternal education was strongly related to the child’s snacking pattern score. Model 1 shows a 0.946 SD higher snacking pattern score (95% CI 0.829; 1.064) for children from low educated mothers and a 0.438 SD (95% CI 0.352; 0.524) higher snacking pattern score for children from middle-high educated mothers compared to children from high educated mothers. After adding paternal education to the model (Model 2), the association between maternal education and snacking score remained strong. Adding household finance and neighbourhood SES (resp. Model 3 and 4) also had a minimal influence on the association between maternal education and snacking pattern scores. Although paternal education, household finance and neighbourhood SES were all significantly associated with the snacking pattern score individually (Table 2), these factors did not overrule the strong association between maternal education and snacking pattern score. Adding all SES factors to the model simultaneously (Model 5) showed similar results.

**Table 2:** Associations between maternal education, different SES indicators and the snacking pattern score (n = 2782).

		B (95%-CI)				
	Maternal education		Paternal education		Household finance	Neighbourhood SES
	Low	middle-high	Low	middle-high	Low	continuously
Model 1: maternal education	0.946 (0.829; 1.064)	0.438 (0.352; 0.524)				
Model 2: paternal education	0.725 (0.547; 0.903)	0.292 (0.183; 0.401)	0.359 (0.198; 0.519)	0.216 (0.112; 0.320)		
Model 3: household finance	0.881 (0.760; 1.002)	0.416 (0.329; 0.503)			0.141 (0.050; 0.231)	
Model 4: neighbourhood SES	0.874 (0.754; 0.994)	0.406 (0.267; 0.479)				-0.085 (-0.113; -0.057)
Model 5: all SES factors	0.673 (0.491; 0.855)	0.283 (0.173; 0.393)	0.304 (0.142; 0.466)	0.199 (0.093; 0.304)	0.108 (0.022; 0.203)	-0.045 (-0.079; -0.011)

Maternal education was based on the highest education completed. Values are based on multivariable linear regression and reflect differences (95% CI) in socio economic factors (maternal education, paternal education, household finance and neighbourhood SES) on the snacking pattern score. Model 1: describes the association between maternal education and the snacking pattern score (n = 2766). Model 2: describes the association between maternal education, paternal education and the snacking pattern score (n = 1799). Model 3: describes the association between maternal education, household finance and the snacking pattern score (n = 2736). Model 4: describes the association between maternal education, neighbourhood SES and the snacking pattern score (n = 2738). Model 5: describes the association between all SES factors and the snacking pattern score (n = 1765). All models were adjusted for age, sex and ethnicity.

### Effect modification between maternal education and household finance

We observed that the role of middle-high maternal education on the snacking pattern score was somewhat moderated by low household finance (p = 0.089), indicating a difference in the association between maternal education and finance on the snacking pattern score. Therefore analyses were stratified by household finance and all models were run within the low and high household finance group separately (Table 3). Higher maternal education was used as the reference. In Model 1, the association between maternal education and the snacking pattern remained strong in both groups. Within the low household finance group, children from lower educated mothers had a 0.821 SD (95% CI 0.641; 1.000) higher snacking pattern score and children from middle-high educated mothers had a 0.4.92 SD (95% CI 0.334; 0.651) higher snacking pattern score compared to children from high educated mothers. We also examined the potential effect modification between household finance and the other SES factors (S1 Table). Results of paternal education were similar to those for maternal education, but the association with neighbourhood SES was small and not statistically significant.

**Table 3:** Effect modification between maternal education and other SES factors, stratified by household finance (n = 2736).

		Low household finance		High household finance	
		Maternal education		Maternal education	
		low	middle-high	low	middle-high
Model 1: maternal education	B (95%-CI)	0.821 (0.641; 1.000)	0.492 (0.334; 0.651)	0.982 (0.800; 1.163)	0.361 (0.258; 0.465)
	B	0.298	0.194	0.241	0.152
Model 5: all SES factors	B (95%-CI)	0.503 (0.223; 0.782)	0.303 (0.088; 0.517)	0.900 (0.624; 1.176)	0.254 (0.125; 0.384)
	B	0.182	0.126	0.178	0.107

Maternal education was based on the highest education completed. Values are based on multivariable linear regression and reflect differences (95%-CI) in socio-economic factors (maternal education, paternal education and neighbourhood SES) on the snacking pattern score. Model 1: describes the association between maternal education and the snacking pattern score in the low and high household finance group (n = 2736). Model 5: describes the association between maternal education, paternal education and neighbourhood SES and the snacking pattern scores in the low and high household finance group (n = 1765). Both models were adjusted for age, sex and ethnicity.

## Discussion

We aimed to examine whether the role of maternal education in children’s dietary patterns is modified by other SES indicators. Our results showed that of all SES indicators, maternal education was most strongly associated with children’s snacking patterns. Moreover, it was independent of other SES indicators and was not moderated by other SES factors studied except for household finance. We found higher snacking pattern scores in children from middle-high educated mothers when household finance was low.

In this study, we used SES indicators commonly used in public health research that are related to dietary patterns, i.e. maternal education, paternal education, household finance and, less frequently studied, neighbourhood SES. Our study corroborates the findings of studies that have considered a range of SES factors in relation to unfavourable dietary patterns in young children. Maternal education, paternal education and household finance (individually or in combination with each other) have been associated with unfavourable dietary patterns [5–7,23–26]. Also in line with our results, studies demonstrated that the influence of household finance on dietary patterns was less pronounced than that of maternal and paternal education [7,27–29]. Furthermore, studies in two other cohorts have observed that maternal education was a stronger determinant of children’s dietary patterns than other SES factors [5,26,30,31]. Only a few studies have considered the role of neighbourhood SES. A Canadian study found higher diet quality in adolescents living in high versus low SES neighbourhoods [10]. An Australian study [8], found a minimal role for neighbourhood SES on dietary patterns. Within the ABCD cohort, earlier results showed that adiposity at age 5-6y was associated with neighbourhood characteristics [32] but that study did not investigate the role of dietary intake. None of the studies mentioned above explicitly investigated whether the role of maternal education on the dietary pattern of young children was moderated by other SES factors. Béghin et al observed that cultural and geographical factors could attenuate the relationship between paternal education and adolescents’ diet quality [26], indicating a potential moderating role of other SES factors. In our study, maternal education was the strongest indicator for an unfavourable snacking pattern, but we also observed a moderating effect of household finance. Higher snacking pattern scores were found in children from middle-high educated mothers when household finance was low.

### Interpretations of findings

The associations between different SES indicators and unhealthy dietary patterns indicate distinct pathways of influence. First, higher educated parents may have higher nutritional knowledge and may perceive long term consequences of diet on the health of their children [33], which is associated with healthier food purchasing leading to healthier dietary patterns of their children [34]. Potential explanations are maternal nutrition knowledge; positive concern and attitudes of the mother towards healthy eating [33,35], self-efficacy, feeding practices and modelling, as well as the quality of foods made available and accessible at home [36]. Knowledge and skills attained through education may affect a person's cognitive functioning, making them more receptive to health education messages, or more able to communicate with and access appropriate health services [3]. Parents, and in particular the mother is considered to take the role of gate-keeper that decides which food items are present in the house and on the table [37,38]. In the HELENA study, results from the Healthy Eating Questionnaire showed that mother-child communication was more effective than father-child communication. Mother was the family member most likely to promote healthy dietary habits [26,39].

Second, household finance reflects the availability of economic and material resources and reflect the potential to purchase a healthy diet [3,4]. Possible determinants are the lower cost per mega joule of energy dense and nutrient poor diets and the easy physical access to low-cost energy-dense foods [40]. Other suggested mechanisms are access to health services and indirect to education, promoting self- esteem and social standing [3]. In the group of middle-high educated women, economic recourses are perhaps a stronger limiting factor than knowledge and attitudes.

Finally, studies in the US and Australia, have shown that the foods available in low SES neighbourhoods are of lower quality, cost more, and have less variety, than foods available in higher SES neighbourhoods [10,11]. At the age of 5, familial factors probably play a more important role in influencing dietary patterns than neighbourhood SES [41], but it’s possible that a poor food environment might influence children through its effect on their parents. Indeed we found an independent association between neighbourhood SES and unhealthy snacking pattern. However, we also found that the association between maternal education and the unhealthy snacking pattern was not moderated by neighbourhood SES levels, implying that maternal education (and the underlying mechanisms) may provide some resistance to challenges presented by a low SES neighbourhood.

### Strength and limitations

A major strength of this study is the ability to examine whether the role of maternal education in young children’s diet is moderated by other SES indicators. Additionally, this study is one of the few studies taking into account the potential role of neighbourhood SES on unfavourable dietary patterns in young children, in combination with more commonly used SES indicators as parental education and household finance. Studying the role of these SES indicators individually, simultaneously and the possible moderating effect of these indicators allowed specific relationships to be highlighted according to the differences in SES. Neighbourhood SES was calculated based on postal codes where children were living at age 5, when most SES factors and dietary intake were measured.

Several limitations should be taken into account. A possible limitation is that we did not have exact data on how long children had been living in their current neighbourhood. It could have been possible that a family moved to a neighbourhood only recently and was living in a neighbourhood with a different neighbourhood SES before. Our study population (mean neighbourhood status score 0.414 (1.18)), may not be representative of children in Amsterdam (-0.282 (1.458)), or other main cities in the Netherlands (-0.969 (1.573) in Rotterdam and 0.0069 (1.705) in the Hague), indicating higher SES of neighbourhoods in our study population. Neighbourhood SES was calculated by the SCP based on 4-digit postal codes. Using 6-digit postal codes would provide more specific information about actual neighbourhood SES, especially for children living at transitional areas of neighbourhoods [42]. However to our knowledge, no appropriate calculation of status score based on 6-digit postal codes was available for our population.

A multilevel approach for data analysis could be considered, given the hierarchical nature of studying how neighbourhood's SES may affect snacking pattern scores in children. By using multiple linear regression analysis instead, it might be possible that the assumption of independence were violated and the generalization of the research findings are limited. 388 different 4-digit postal codes were included in our analysis and the mean number of participants per postal code was 7.1 (13.6). To ensure sufficient power for statistical analysis, postal code areas with less than 10 children would have been excluded to ensure sufficient power for statistical analysis [32].

Paternal education was collected during the 10-year questionnaire and the number of missing values was substantial (n = 979). Compared to children with complete data, children with missing data on paternal education had more often lower educated mothers (resp. 6.4% and 17.5%), lower household finance (resp. 27.4% and 42.3%), lower neighbourhood SES (resp. 0.414 (1.179) and -0.038 (1.344)) and higher snacking pattern scores (-0.146 (0.902) and 0.268 (1.109)). However, sensitivity analyses within children with complete data on dietary patterns and all SES factors and potential confounders (n = 1765) showed similar results.

Attrition of study population was largely due to untraceable changes in address or migration. A nonresponse analysis determining the degree of selective response and selection bias between pregnancy and birth outcomes, indicated that selective non-response was present in the ABCD-study, but selection bias was acceptably low and did not influence the studied birth outcomes [43]. No further nonresponse analysis was done. It is possible that some biases may have been introduced into the analyses, particularly as the non-responders tended to come disproportionately from lower SES and ethnic minority groups.

### Implications

We found that all SES indicators were independently related to snacking pattern scores of children age 5 and we observed a cumulative association of these different SES indicators that add to the risk of an unhealthy dietary pattern. However, the influence of maternal education remained strong, despite addition of other SES indicators. Lower snacking pattern scores were found in children of middle-high educated woman with sufficient household income. This suggests that primary focus for intervention strategies should be given to lower educated mothers, with particularly targeting of mothers were more unfavourable SES indicators are prevalent and middle-high educated mothers with insufficient levels of household finance.

## Conclusions

Our results show that all SES indicators contribute to the risk of unhealthy dietary patterns in a population of young children. Low paternal education, lower household finance and lower neighbourhood SES were all independently related to higher snacking pattern scores, but they did not affect the strong relationship between low maternal education and the intake of a dietary pattern characterized by snack foods. Within the group of middle educated mothers, higher snacking patterns were found when the level of household finance was low.

## Supporting information

S1 Table Interaction between maternal education and other SES factors on the snacking pattern score (n = 2782). (DOCX)

## List of abbreviations

SES: socio-economic status
FFQ: Food Frequency Questionnaire
ABCD: Amsterdam Born Children and their Development
SCP: Social and Cultural Plan Bureau
PCA: Principal Component Analysis
SD: standard deviations
ALSPAC: The Avon Longitudinal Study of Parents and Children
