# Supplementary material for: Beyond maternal education: Socio-economic inequalities in children’s diet in the ABCD cohort
Source: PLoS One. 2020 Oct 13;15(10):e0240423. doi: 10.1371/journal.pone.0240423 (PMC7553270; doi:10.1371/journal.pone.0240423)
Supplement: S1 Table — (DOCX) [file pone.0240423.s001.docx]

**S1 Table. Interaction between maternal education and other SES factors and the snacking pattern score (n=2782).**

|  |  | Maternal education | | Paternal education | | Neighbourhood SES |
| --- | --- | --- | --- | --- | --- | --- |
|  |  | low | middle | low | middle | continuously |
| Low household finance |  |  |  |  |  |  |
| Model 1: | B (95%-CI) | 0.821 (0.641; 1.000) | 0.492 (0.334; 0.651) |  |  |  |
|  | β | 0.298 | 0.194 |  |  |  |
| Model 5: all SES factors | B (95%-CI) | 0.503 (0.223; 0.782) | 0.303 (0.088; 0.517) | 0.314 (0.050; 0.578) | 0.345 (0.140; 0.551) | -0.069 (-0.114; 0.003) |
|  | β | 0.182 | 0.126 | 0.113 | 0.147 | -0.083 |
| High household finance |  |  |  |  |  |  |
| Model 1: | B (95%-CI) | 0.982 (0.800; 1.163) | 0.361 (0.258; 0.465) |  |  |  |
|  | β | 0.241 | 0.152 |  |  |  |
| Model 5: all SES factors | B (95%-CI) | 0.900 (0.624; 1.176) | 0.254 (0.125; 0.384) | 0.326 (0.109; 0.542) | 0.117 (-0.006; 0.240) | -0.036 (-0.075; 0.003) |
|  | β | 0.178 | 0.107 | 0.084 | 0.052 | -0.049 |

Maternal education was based on the highest education completed. Values are based on multivariable linear regression and reflect differences (95% CI) in socio-economic factors (maternal education, paternal education and neighbourhood SES) on the snacking pattern score. Model 1: describes the association between maternal education and the snacking pattern score in the low and high household finance group (n=2736). Model 5: describes the association between maternal education, paternal education, neighbourhood SES and the snacking pattern score in the low and high household finance group (n=1765). Both models were adjusted for age, sex and ethnicity.
